# From seed to shoot: the organ-specific composition of the vicine/convicine pool in faba bean

**DOI:** 10.3389/fpls.2026.1923913

**Published:** 2026-10-28

**Authors:** Kjell Sergeant, Marion Barczyk, Hanna Heidt, Hanna Tietgen, Stéphanie Zimmer, Salma Halime, Cédric Jacquard, Jenny Renaut

**Affiliations:** 1 Plant Molecular Farming, Environmental and Industrial Biotechnologies, Luxembourg Institute of Science and Technology, Hautcharage, Luxembourg; 2 IBLA – Institut fir Biologesch Landwirtschaft an Agrarökologie Luxemburg a.s.b.l., Medernach, Luxembourg; 3 NPZ Innovation GmbH, Holtsee, Germany; 4 Université de Reims Champagne-Ardenne, INRAE, Research Unit RIBP UMR 1488, Reims, France

**Keywords:** derivatives, LC-MS/MS, metabolomics, *Vicia faba*, vicine and convicine

## Abstract

Divicine and isouramil, the aglycons of vicine (VC) and convicine (CV) pyrimidine glycosides present in faba bean (*Vicia faba* L.) seeds, cause favism in individuals with glucose-6-phosphate dehydrogenase deficiency. Although breeding has resulted in low vicine and convicine (V-CV) cultivars, recent metabolomic studies described V-CV derivatives whose biological relevance and distribution across plant organs are unknown. Here, the composition of the V-CV derivative pool in seeds and vegetative organs during early seedling development is described. Metabolic profiling was performed on seeds of 10 faba bean cultivars representing genotypes having or lacking the low -VC (LVC) trait. cultivars lacking the low-VC trait, have a normal content in VC and CV and are annotated here as high-vicine convicine (HVC) cultivars. In addition, the V-CV pools of seeds, germinated seeds, roots, and shoots were compared for one variety having the LVC trait and one lacking this trait. Seed profiling revealed a seed-specific derivative pool, and although most of the derivatives showed lower signal intensities in LVC cultivars, cultivar-specific fingerprints were observed independent of the abundance of V-CV. The composition of the V-CV pool indifferent organs changed from relatively simple seed-associated compounds toward chemically complex derivatives in the roots and shoots. Principal component analysis (PCA) and hierarchical clustering showed organ identity as the primary determinant of the V-CV pool composition, with genotype as the secondary factor. Our data on the seeds indicates that genetic background influences both the precursor availability and derivatization pathways. The comparison of the V-CV profiles in the different organs suggests that V-CV metabolism involves active, organ-specific derivatization processes. Given the diversity and high MS signal intensities for some derivatives, the inclusion of these in studies on the biosynthesis, distribution, and toxicity of V-CV may result in new insights.

## 1 Introduction

Members of the Fabaceae family of plants are of interest as sources of food and feed and for medical purposes. The entire pods or seeds from species such as soybean, peas, faba bean, lentils, and chickpeas are an essential part of local cuisines and are currently consumed worldwide. With a protein content two- to threefold higher than cereals (Jha et al., 2022), international efforts such as the European Protein Initiative aim to promote the cultivation of these low-input protein-rich crops as an alternative to animal protein. This agronomic interest is tempered by protein and non-protein anti-nutritional compounds (Sammoud et al., 2026). Among the non-protein anti-nutritional compounds, saponins and phytates occur across a broad range of Fabaceae seeds. Other metabolites found in seeds of a limited number of species are toxic either for the general public or for susceptible consumers. Neurolathyrism, which is caused by the consumption of seeds from the grass pea (*Lathyrus sativus* L.), is an example of the first category (Lambein et al., 2019), as are the neurotoxic effects of gamma-glutamyl-beta-cyanoalanine synthesized by *Vicia sativa* (Roy et al., 1996). Favism, which is caused by the consumption of faba beans (*Vicia faba*) in individuals with glucose 6-phosphate dehydrogenase (G6PD) deficiency (Beretta et al., 2023), is an example of the latter category.

Favism is caused by the aminopyrimidines divicine and isouramil, which are generated from the glycoalkaloids vicine (VC) and convicine (CV) after hydrolysis of the glucose moiety. *In planta* vicine and convicine, referred to as V-CV when both compounds are concerned, contribute to the defense against herbivores and, although supported by only a few studies, have a reported antifungal activity (Bjerg et al., 1984; Pavlík et al., 2002). With G6PD deficiency being the most prevalent enzymopathy in the world affecting approximately 6% of the population (Beretta et al., 2023), a low V-CV content is an important breeding target in faba bean (Khazaei et al., 2019; Schlichtermann et al., 2025), and low V-CV varieties have been developed (Adhikari et al., 2021). Although no low-VC (LVC) were used and only VC was quantified, a recent study on 189 faba bean varieties showed a positive correlation between VC content and grain yield. Based on this, the authors of this study concluded that the development of near-zero VC faba bean may drastically reduce yields and that cautious and efficient selection strategies must be followed (Debnath et al., 2025). The interest in these compounds has led to the development of targeted analytical methods for their absolute quantification, which are now in routine use (Purves et al., 2018).

These quantification methods specifically target the compounds VC and CV. Nonetheless, derivatives of V-CV were described, and for 89 derivatives observed in the roots and shoots of plantlets from different faba bean varieties, a tentative identification was proposed (Sergeant et al., 2024). For other compounds, the presence of a V-CV group could be confirmed, but with no tentative identification proposed. Given the anti-nutritional properties of V-CV and the possibility that, after digestion, the described derivatives contribute to the actual amount of divicine and/or isouramil to which an organism is exposed, further study is needed. As such, a better approximation of the actual toxicity of faba beans can be established. This is a toxicity that, although bioassays are available (Getachew et al., 2018) and no clear-cut thresholds exist, is currently assessed through the quantification of VC and CV, the mother compounds of the derivatives described here, alone. The identification of these derivatives furthermore raises questions about the origin of V-CV in different plant organs. The expression of the genes coding for VC1, the enzyme that catalyzes a key step in VC biosynthesis (Björnsdotter et al., 2021), is mainly limited to the seed coats (Ugwuanyi et al., 2025b), a maternal tissue. Further study indicates that the VC content is determined by single-copy expression in a multi-copy number genetic background (Ugwuanyi et al., 2025a). However, older data, which showed that the amount of V-CV in seeds is not sufficient or requires VC-to-CV conversion to account for all V-CV found in plants (Griffiths and Ramsay, 1996; Ramsay and Griffiths, 1996), suggest that these compounds are also synthesized in other organs. The composition of the V-CV pool, i.e., all compounds that contain a VC or a CV, of the different organs and the factors that determine this thus warrant further study.

The result of our previous study raises two questions that this work addresses. Because seeds are the organs that are effectively consumed and since some V-CV derivatives have already been described in seeds (Valente et al., 2019; Kowalczyk et al., 2021; Irmer et al., 2026), a first question is on the composition of the V-CV pool of seeds. Related to this is the question of whether the V-CV pool is determined by the genetic capacity of the different genotypes to synthesize the mother compounds. Second, how does the composition of this pool evolve from seeds to vegetative organs? Based on our previous study, we hypothesize that the V-CV pool has a structured organ- and genotype-specific composition shaped by localized metabolism and, thus, genetic background.

To test this, the V-CV profile of the seeds of 10 cultivars, which cover genotypes having a LVC trait and genotypes lacking this trait, with a normal content and annotated here as high-vicine convicine (HVC) cultivars was established. Furthermore, the composition of the V-CV pool of the seeds, germinated seeds, plantlet roots, and shoots was compared for one LVC cultivar (Allison) and one HVC cultivar (Trumpet). To do this, the newly acquired seed data were combined with previously reported vegetative tissue profiles (Sergeant et al., 2024). To account for matrix-dependent differences in the extraction efficiency across organs, derivative abundances were expressed as the proportional contribution of each compound to the total V-CV pool rather than as the absolute MS signal intensities.

## 2 Materials and methods

### 2.1 Sampling and extraction

Ungerminated seeds of 10 faba bean varieties, selected to span the LVC/HVC classification usedhere, were provided by the Institute for Organic Agriculture and Agroecology (IBLA, Luxembourg). For extraction from the seeds, a pool of 10 seeds was made for each variety and ground. Because they originated from the same seed batch, this pool can be considered to represent an average for this seed batch, while differences between seed batches of the same variety can result in the modulation of the metabolite profiles. The terms HVC and LVC are defined respectively as faba bean varieties that lack or have the low-VC trait, information that can be found in breeder catalogues and is represented in **Supplementary Table S1**. The 10 varieties included three LVC cultivars (Allison, Callas, and Futura) and seven HVCcultivars (Birgit, Daisy, Fanfare, Macho, Stella, Sunrise, and Trumpet). One HVC variety, Sunrise, is white-flowered and tannin-free. The seed provider, the presence or absence of the LVC trait, and the field performance data during the 2023 season are given in **Supplementary Table S1**. The roots, shoots, and germinated seeds were provided by Norddeutsche Pflanzenzucht KG (NPZ, Holtsee, Germany). Randomly selected seeds from a field-produced seed lot were grown in the NPZ seed quality laboratory using the standard method for ISTA germination. In total, 30 seeds were sown in sand substrate in a plastic box and placed in a climate chamber at 20°C with 16 h under light and 8 h under dark conditions. The boxes were covered with black foil for 7 days, before the foil was removed, and the seedlings were grown for another 7 days, corresponding to the BBCH scale EC10–11 stage (cotyledon—first true leaf). Three biological replicates each composed of 10 plantlets were sampled, frozen, and sent to LIST in Hautcharage, Luxembourg. Shoots, roots, and germinated seeds remaining attached to the seedlings were separated, lyophilized, and ground. One of the germinated seed replicates of Allison got lost during extraction; hence, two replicates of the germinated seeds were analyzed.

The varieties Allison (LVC) and Trumpet (HVC) are common to both datasets and were therefore selected for the seed-to-seedling comparison.

All samples were extracted with the same protocol: ground plant tissues are weighed and 80% MeOH/20% H_2_O MQ added at a 1:12 *w*/*v* ratio. The samples were homogenized by 5 min of sonication (22°C, 100% power, 37 Hz) and subsequently incubated at 24°C and 1,400 rpm to extract metabolites. After 1 h, the samples were centrifuged for 20 min at 4°C and 20,000 × *g*, the supernatant containing the metabolites was collected and transferred into new tubes, and then dried under vacuum and stored at −20°C prior to analysis.

### 2.2 LC-MS/MS analyses and identification

The dried extracts were resolubilized in 5% MeOH/MQ in the same volume used for extraction. After filtering through a 0.22-μm PTFE syringe filter (Millex-LG, Merck KGaA, Darmstadt, Germany), 10 µl of the extracts was separated with an Acquity UPLC I-class system equipped with a diode array detector (Waters, Milford, MA, USA) hyphenated to a TripleTOF 6600 (AB SCIEX, Framingham, MA, USA) mass spectrometer as previously described (Backes et al., 2021). The analytes were separated on a reversed-phase column (Acquity UPLC BEH C18 column, 2.1 mm × 100 mm, 1.7 μm particle size; Waters) at 50°C and at a flow rate of 0.5 ml/min. The eluents were 0.1% (*v*/*v*) formic acid in water (A) and 0.1% (*v*/*v*) formic acid in acetonitrile (B) with the following gradients: 0 min, 1% B; 4 min, 1% B; 16 min, 5% B; 35 min, 40% B; 45 min, 100% B; 50 min, 100% B; 54 min, 1% B; and 60 min, 1% B.

Electrospray ionization (ESI) was performed using the following parameters: source temperature, 650°C; ion spray voltage, 4.5 and −4.5 kV; curtain gas (nitrogen), 30 psi; nebulizer gas (air), 55 psi; and turbine gas (air), 50 psi. The declustering potential was set up at 60 and −60 V in positive and negative modes, respectively. The precursor charge state selection was set at 1. For information-dependent acquisition, MS scans (from *m*/*z* 100 to 2,000) were acquired for 175 ms, and the 10 most abundant *m*/*z* values were selected for tandem mass spectrometry (MS/MS) scans (from *m*/*z* 50 to 2,000) of 200 ms. A sweeping collision energy of 15 V below and above 15 and −15 V, for the positive and negative modes, respectively, was applied to all precursor ions. After three occurrences, an *m*/*z* value was excluded for MS/MS for 2 s.

For the identification and relative quantification, the raw data files were uploaded in Progenesis QI (v2.3; Nonlinear Dynamics, Newcastle upon Tyne, UK). Different Progenesis experiments were created, one for the seeds described in part one, one for the roots, one for the shoots, and one for the germinated seeds. LC-MS features without MS/MS spectrum were filtered out, as were the peaks corresponding to known or suspected contaminants. The remaining features were used for identification. Initially, the data were searched using msp files extracted from our in-house database, among others containing the V-CV derivatives previously reported (Sergeant et al., 2024). Other V-CV derivatives were recognized by filtering the LC data for compounds having characteristic fragment ions. The base peak in the MS/MS spectra of VC is divicine, the aglycone formed by the loss of glucose. The formula of the aglycone is C_4_H_6_N_4_O_2_, resulting in a calculated *m*/*z* value of 141.041 in the negative mode analyses. In the negative mode MS/MS spectra of CV, the base peak is at *m*/*z* 141.0174 and corresponds to deprotonated [isouramil − H]. This corresponds to the use of the fragment at *m*/*z* 141 in the selected reaction monitoring (SRM) analysis of both mother compounds (Purves et al., 2018). To cover derivatives with both mother molecules, compounds resulting in MS/MS spectra containing a peak at 141.03 ± 0.015 were filtered out as previously used by us and others (Valente et al., 2019). For this, the fragment filter tool of Peakview (version 2.2.0.11391; AB SCIEX) was used. False positives were further avoided by manually verifying the presence of an *m*/*z* value corresponding to VC or CV, checking whether the exact mass of the 141-fragment corresponds to that of the mother compound and considering the previously observed neutral loss of 124.04 amu [divicine − H_2_O] from VC derivatives. References to compounds in the text are indicated in bold and refer to **Supplementary Table S2**.

For the semi-targeted identification reported here, a hypothetical identification was proposed after manual spectral interpretation. The structure was generated in Chemdraw (v.23.1.2), and the theoretical MS/MS spectrum of this structure, generated by MetFrag (https://ipb-halle.github.io/MetFrag/) and CFM-ID (https://cfmid.wishartlab.com/) (Ruttkies et al., 2016; Wang et al., 2022), was compared to the experimental spectrum. Accepted identifications were subsequently added to our in-house database for dereplication.

The confidence of each annotation is reported according to the communication scheme of Schymanski et al. (2014). In the absence of authentic standards, as well as the identification of the VC and CV core, as defined above, the elemental composition of the derivatizing group was determined by accurate MS while, when possible, attachment to the V-CV glucose was confirmed. This makes that the derivatives are reported as tentative candidate structures (level 3). The molecular formula, measured mass error, isotope similarity, and fragmentation score supporting each annotation are given in the supplementary tables (median fragmentation score = 87, median absolute mass error = 3.8 ppm, median isotope similarity = 95).

### 2.3 Statistical analysis

Feature alignment, normalization, and semi-quantitative extraction were performed in Progenesis QI (v2.3). Principal component analysis (PCA) was computed in Progenesis QI and MetaboAnalyst 6.0.

PCA and the analysis of the relative abundance of compounds in the comparison of the seeds were done in Progenesis. For this, the embedded normalization option to normalize based on all features was used. Hierarchical clustering in the comparison of the seeds and all statistical analyses for the second part of this study were done in MetaboAnalyst.

For the analysis of the seeds, the data resulted from a single Progenesis analyses, and when a feature is not observed in one of the samples, a value of zero is attributed by default. For PCA, the normalized data extracted from Progenesis were used without further normalization or scaling. For heatmap analysis, each feature was autoscaled and clustering was performed with the Minkowski measures and Ward clustering. For the analysis of the data from the different tissues, data were extracted from the different Progenesis experiments and the raw data values used. A data matrix was composed of all tentative identifications obtained in the different experiments. When a compound was not identified in one of the organs, the value of zero was attributed, as is done in Progenesis should all datasets have been analyzed in one experiment. The sum of the raw intensity of all features from each sample was calculated and the values for each feature divided by this sum. The thus obtained value was multiplied by 1,000 to attain the promille contribution of each compound to the total V-CV pool of each sample. The obtained data matrix was uploaded to MetaboAnalyst, and the autoscaled data were used for the generation of a heatmap using the Minkowski measures with complete clustering. The PCA was generated without further normalization or scaling using the PCA function of scikit-learn (version 1.9.0) in Python (version 3.14.4), with NumPy (version 2.4.4) and pandas (version 3.0.2) for data handling.

## 3 Results and discussion

### 3.1 V-CV profiling of faba seeds

The seeds of 10 varieties of faba bean were extracted and analyzed with LC-MS/MS. After importing in Progenesis, eliminating known contaminants and LC features without MS/MS, 2,824 features remained. PCA of these 2,824 features showed that, in this dataset, the varieties classified as LVC clustered together, while varieties classified as HVC showed a higher variation (**Supplementary Figure S1**). This clustering of varieties based on their classification as LVC or HVC indicates that the genetic base used for the generation of the LVC varieties is the dominant factor, with repercussions beyond the V-CV content.

Compared with the V-CV derivatives profiled in the roots and shoots, new derivatives were added, with some of these having been reported in other studies focused on the phytochemical profile of seeds. These are, among others, CV hexoside (compound **5**) and compounds 61 and 25 (Tuccillo et al., 2025). The structurally characterized V-CV derivatives described by Imer et al. were likewise found in our dataset (Irmer et al., 2026). While our previous profiling of the shoots and roots did not allow identifying all the V-CV derivatives identified in previous studies (Valente et al., 2019; Kowalczyk et al., 2021; Karolkowski et al., 2023), they were identified in the seeds, confirming that seeds contain a unique set of V-CV derivatives.

The general observations previously made for the V-CV derivatives identified in the roots and shoots remain valid. The CV form of a derivative generally elutes earlier than the VC form, and VC derivatives often undergo an in-source loss of 124.039 amu [divicine − H_2_O], while no such in-source fragmentation is observed for CV derivatives. For compounds formed through the linkage of a VC and a CV, an intense peak at *m*/*z* 141.04 (indicative of VC) is proceeded by a small but distinguishable fragment peak at *m*/*z* 141.018 (corresponding to deprotonated uramil − H). Several of the structures proposed here have been confirmed independently using NMR on compounds isolated from faba bean. Irmer et al. (2026) reported VC- and CV-6-*O*-isovalerate, in which the isovaleryl group is esterified at the 6-position of the glucose, together with the diglucosides and glucosyl-xylosides of divicine and isouramil (Irmer et al., 2026). These correspond, respectively, to the (iso)valeryl, hexoside, and pentoside derivatives annotated here, resolving the pentose as xylose and the second hexose as glucose. This independent structural work confirms these specific annotations and supports the general observation that the derivatizing groups are borne on the V-CV glucose rather than on the aminopyrimidine.

After elimination of dimers and in-source fragments, 104 LC-MS features containing a V-CV were retained (**Supplementary Table S2**). This number is lower than that of the derivatives found in roots and shoots, and the V-CV-profile of seeds is more dominated by relatively simple compounds. For instance, only seven compounds formed by the linkage of one V-CV to another, with a linker formed by a C_10_H_12_O_3_ or a C_12_H_14_O_4_ or a C_12_H_16_O_4_, were identified. V-CV pentoside (compounds 4 and 6), the VC form of which was reported in 2021 (Kowalczyk et al., 2021), are among the high-MS signal intensity derivatives not identified in the roots or shoots. The first principal component of a PCA with these 104 V-CV derivatives separated the LVC from the HVC varieties with the variety Daisy intermediate (**Figure 1**).

**Figure 1:**
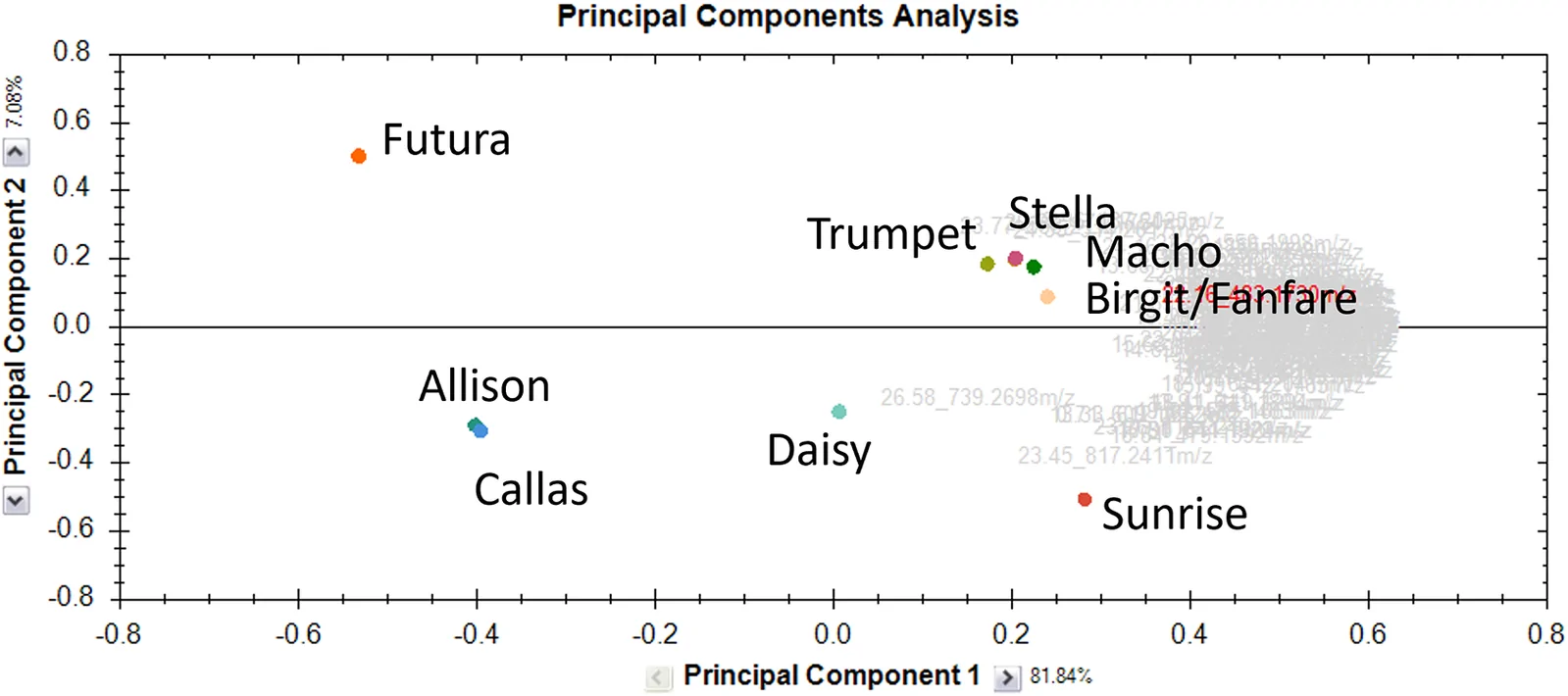
Principal component analysis (PCA) of the 104 tentatively identified vicine and convicine (V-CV) derivatives based on the MS signal intensity data from 10 varieties of faba bean. The low-VC (LVC) varieties Futura, Allison, and Callas are separated from the high-VC (HVC) varieties on PC1, representing 82% of the variation in the dataset. Daisy, formally a HVC, is found in between these two groups.

A first observation is that the MS signal intensity of CV (compound **2**) was higher than that of VC (compound 1) for all varieties. The current study is based on untargeted LC-MS analysis, which allows identifying all derivatives, but is a semi-quantitative approach. Based on absolute quantification with HPLC or dedicated LC-MS analyses, the ratios of VC to CV reported in the literature are generally between 1.8 and 3.5 (Pulkkinen et al., 2015), although higher ratios have been reported (Ramsay and Griffiths, 1996). Differences in ionization efficiency can contribute to the observation that, in our dataset, this ratio is below 1 for all varieties. A more important factor is probably that VC and CV coelute and that there is a contribution of the second isotope of VC to the signal intensity of the first isotope of CV. Given the semi-quantitative nature of the data, no comparison of the abundance rates between the different compounds can be made and only the abundance, represented as the MS signal intensity, of the same compound in different varieties can be compared.

All but three of the 104 V-CV derivatives had the highest intensity values in the HVC varieties.The relative MS signal intensity of the majority of derivatives is thus positively correlated with the LVC/HVC classification as defined in “*Methods*” (**Supplementary Table S1**). This correlation is maintained considering only the signal intensity of the mother compounds (compounds 1 and 2) (**Supplementary Table S2**). However, comparison of the signal intensities of the mother compounds with those of the derivatives gives a first indication that the abundance of VC and CV is not the only factor that determines the abundance of derivatives. Stella had the highest MS intensity values for VC and CV, but for only four more derivatives. This indicates that high intensity, and thus relative abundance for the mother compounds, is not directly related to the high MS signal intensities for derivatives and that other genetic factors also contribute.

These 104 derivatives were used for statistical analysis. Hierarchical clustering in MetaboAnalyst 6 (Pang et al., 2024) confirmed that Daisy is indeed close to the LVC group (**Figure 2**). In general, CV and VC with the same derivatization are in the same cluster, which may indicate that derivatization is not primarily dependent on the glycoalkaloid.

**Figure 2:**
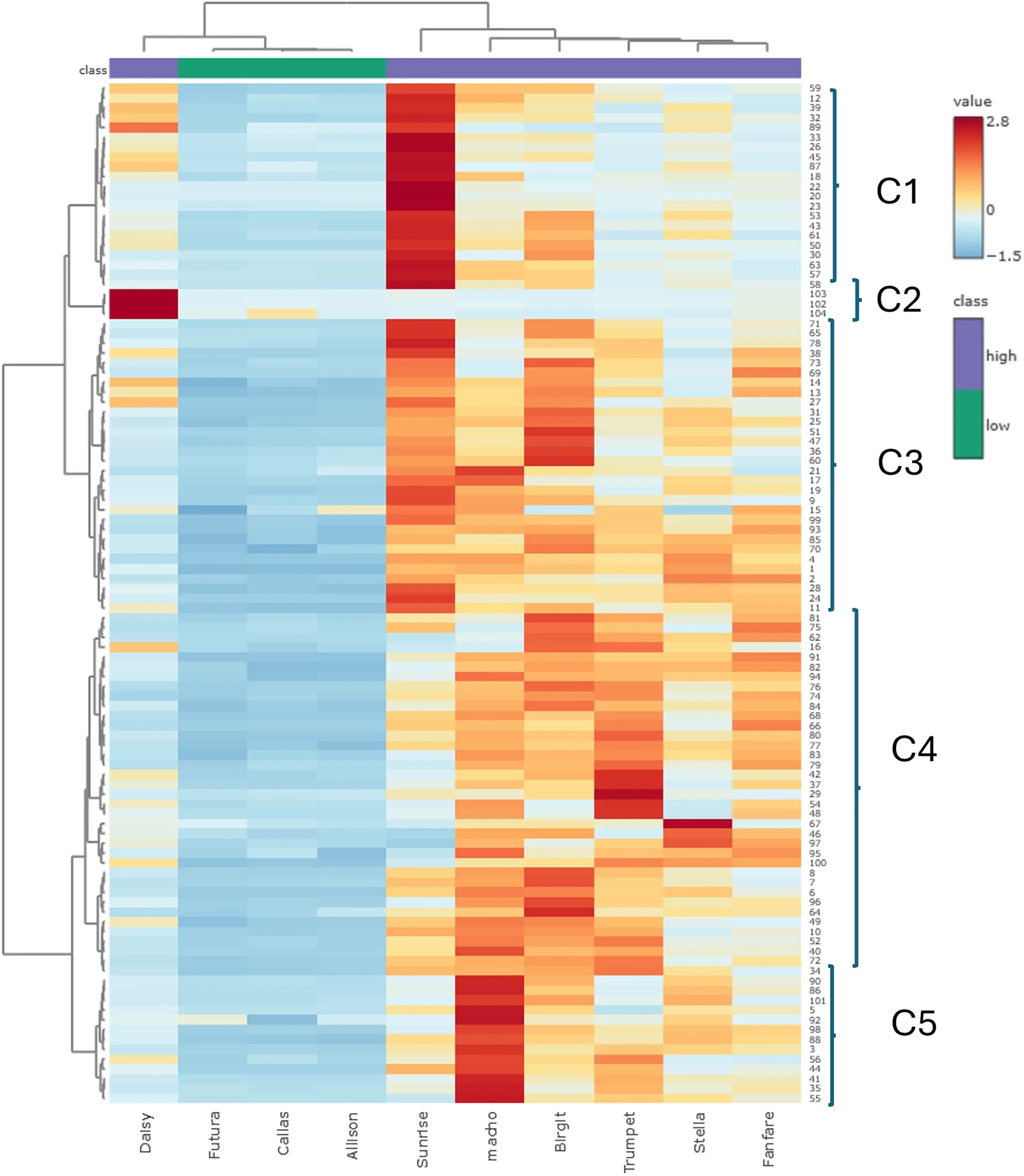
Heatmap analysis of the 104 tentatively identified vicine and convicine (V-CV) derivatives basedon the MS signal intensity data. Low-VC (LVC) varieties (indicated *above the heatmap in green*) form a group with low intensity for all identified derivatives. The high-VC (HVC) varieties (indicated in *purple*) also form a group, with the exception of the variety Daisy that has a low content for the majority of derivatives, but contains three derivatives that are absent in the other varieties (C2). The tentative identifications of the references given on the *right side of the heatmap* can be found in **Supplementary Table S2**.

The first cluster (C1; **Figure 2**) contains 21 derivatives that are most abundant in Sunrise. Of the 28 identified derivatives containing a hydroxycinnamic acid (HCA) (coumaric, caffeic, ferulic, or sinapinic acid), 20 were found in this cluster. Looking at the total metabolic profile of the seeds, the coumaric and ferulic acids and the mono- and dihexosides of these are also highest in Sunrise seeds, the only tannin-free, white-flowered variety in the test set. While no independent phenolic profile for the varieties used here is available, the differences in the accumulation of targeted phenolic compounds has been assessed in other studies (Johnson et al., 2021). Contrary to the variety PBA Rana in the latter study, the high abundance of HCA glucosides in Sunrise does not coincide with a higher abundance of other phenolic compounds. Prodelphinidin, (epi)(gallo)catechin, and procyanidins had the lowest MS signal intensity in Sunrise (**Supplementary Table S3**), while majority of the identified phenolic compounds showed maximum intensity in the variety Macho. These lower values for majority of the phenolics, HCAs excluded, agree with previous data showing the variety Sunrise to have the second lowest total phenolic content value out of 15 tested varieties (Mayer Labba et al., 2021). The elevated HCA content and the depleted flavonoid profile of Sunrise are consistent with its white-flowered, acyanic phenotype. White coloration in flowering plants commonly results from enzymatic blocks within the flavonoid biosynthesis pathway, during steps including flavanone 3-hydroxylase, dihydroflavonol 4-reductase, or anthocyanidin synthase. Such blockage would redirect the phenylpropanoid flux toward HCA accumulation rather than downstream pigment production (Mato et al., 2000).

The most striking observation concerns C2 containing compounds 102, 103, and 104, the most hydrophobic compounds identified. These derivatives, VC derivatized with C_14_H_18_O_3_ and C_14_H_16_O_3_ with a (malonyl)hexose, are close to unique in the variety Daisy. Although Daisy is classified here as HVC, or on the supplier website as having a normal VC content, the MS signal intensities of the mother compounds are also intermediate between that of the LVC and HVC varieties (**Supplementary Table S2**). Since the current data are based on one replicate, which was extracted from a pool of seeds from one batch of seeds, this observation may be specific for this batch of seeds and not representative for others. While this requires confirmation, also excluding the highly specific profile of Daisy does not eliminate the observation that each variety has a specific V-CV pool. To try to understand this, the LC data were searched for compounds with chemical formulas C_14_H_20_O_4_ and C_14_H_18_O_4_, the derivatizing groups with added water, that are of relatively higher abundance in Daisy; however, no candidates were found. A literature survey of compounds known in Fabaceae likewise did not result in discerning candidates.

C3 (**Figure 2**) contains the derivatives with high intensity values in Sunrise and Birgit. The mother molecules are in this cluster, as are the derivatives annotated as (iso)valeryl V-CV that had the highest MS signal of all derivatives in all varieties. In this cluster, there were six derivatives containing a HCA, two of which, again, had the highest values in Sunrise. The compounds CV coumaroyl (hexoside) and VC caffeoyl (hexoside) were highest in Macho, while VC sinapoyl (malonyl) hexosides (compounds 36, 60, and 64) were highest in Birgit. However, the differences in the MS signal intensities for these six HCA derivatives between the HVC varieties were small compared with those of the HCA derivatives in cluster 1.

Majority of the V-CV derivatives in C4 had maximum values in the varieties Stella, Trumpet, and Birgit. The compounds highest in Trumpet were VC and CV linked to (iso)valeryl hexoside, C_12_H_16_O_3_ hexoside, and C_12_H_16_O_4_ hexoside. Furthermore, a group of three compounds formed by a linkage of two V-CV molecules with C_12_H_16_O_4_ (compounds 66, 68, and 72) were in this cluster. The derivatives that were highest in Birgit are, again, pairs of VC and CV derivatized with the same group. A total of 10 compounds that contained C_12_H_16_O_4_ were identified, and hexoses of this derivative were highest in Sunrise (cluster 3). For the seven compounds containing this group in C4, the lowest values were obtained for Stella and Sunrise.

C5 contained V-CV derivatives that had maximum values in Macho. Except for V-CV hexoside (compound 3 and 5), all derivatives possessed what we hypothesize to be dicarboxylic groups, eluting at retention times greater than 18 min.

Our data showed that the abundance of V-CV derivatives in the seeds of the different varieties depends on multiple factors, and the seeds of the 10 varieties each have a specific V-CV profile. The abundance of the mother compounds was not the only factor determining the abundance of derivatives. The highest MS signal intensity for the mother compounds was found in Stella; however, a few of the V-CV derivatives reached a maximum in this variety. Nonetheless, for all compounds, except for compounds 102–104, there was a clear correlation of this parameter at least in the distinction between HVC and LVC varieties. While precursor supply is thus a factor that sets the great lines, specific enzymatic activities that catalyze the derivatization or the availability of derivatizing groups, as observed for Sunrise, determine the final composition of the V-CV pool in seeds.

For the variety Sunrise, a high abundance of HCA V-CV derivatives is concomitant with a high abundance of the derivatizing groups in the metabolome of this variety, but with no such correlation found for the other derivatizing groups. However, precursor availability could be a determinant, thereby potentially linking the V-CV profile with the genetic regulation of other pathways. Furthermore, the co-clustering of pairs of V-CV derivatives bearing the same group suggests that the conjugating enzymes show little selectivity for the aglycone, consistent with the absence of derivatization at the pyrimidine reported above.

### 3.2 Composition of the V-CV pool from seed to seedling

In the second part of this study, the change in the composition of the V-CV pool from seed toseedling was followed for two faba bean varieties: Allison, a LVC, and Trumpet, a HVC (**Supplementary Table S1**). For this, data from the seeds presented above were combined with data from our previous study (Sergeant et al., 2024) and were reanalyzed using the expanded in-house database. This grouping of data from different organs, which were acquired from different batches and analyzed in different Progenesis experiments, necessitated the use of a specific analytical approach. This allows only the comparison of the contribution of the derivatives to the V-CV pool in the different organs, while a discussion on the relative abundance of a derivative in the different organs is not possible.

To confirm that the obtained metabolite profiles for seeds and germinated seeds correspond to the current knowledge on the metabolome of these organs, the data were compared with published data on key metabolites. While ungerminated seeds contain high amounts of the oligo-hexoses stachyose, raffinose, and verbascose, already after 3 days of germination, the content of these goes to zero (Wei et al., 2022). The same decrease in these compounds was observed in our data comparing seeds and germinated seeds, thereby validating that our data are coherent with current knowledge. Although we did not identify l-DOPA, several l-DOPA derivatives were identified. These included feruloyl l-DOPA, previously identified in *Trifolium pallidum* (Szajwaj et al., 2011), glycosides of this, and l-DOPA hexoside (Nagasawa et al., 1961). These l-DOPA derivatives increased between seeds and germinated seeds, as was noted for l-DOPA in other studies (Goyoaga et al., 2008; Wei et al., 2022). Another study identified four isoforms of l-DOPA hexoside, with different accumulation patterns dependent on the variety (Mekky et al., 2020). Furthermore, the main flavonoids in our extracts had accumulation profiles as previously reported. Isorhamnetin and apigenin glycosides, which are abundant in faba sprouts (Mekky et al., 2020), were not identified in the seeds, while quercetin, kaempferol, luteolin, and myricetin glycosides were identified (Kwon et al., 2018; Duan et al., 2025). In general, metabolomic observations on the seeds and germinated seeds correspond to those of previous studies.

Although the same extraction protocol and identical analytical parameters were used, direct semi-quantitative comparison based on the MS signal intensity is impossible. Because the characteristics of the plant materials are too different, differences in the extraction efficiency would result in biases in the interpretation. Therefore, the raw MS signal intensity values of the V-CV derivatives were extracted from Progenesis QI and the *per mille* contribution of each derivative to the V-CV pool calculated. This value was obtained by dividing the raw MS intensity value of each derivative (represented in **Supplementary Table S4**) by the sum of the raw MS intensity of all derivatives in a sample and multiplying this with 1,000. After the elimination of dimers and in-source fragments, 160 V-CV derivatives were retained.

PCA separates the seeds, germinated or not, from the vegetative tissues on PC1, with the ungerminated seeds separated from the germinated seeds on PC2. The vegetative tissues were also separated on PC2, with a close clustering for the shoot samples of both varieties, while the variability between and within varieties of the root samples was higher (**Figure 3**). Hierarchal clustering, using complete clustering with Minkowski measures in MetaboAnalyst, confirmed that the main discriminant in the dataset is the organ (**Supplementary Figure S2** and **Supplementary Table S4**). Seven clusters can be discerned. C1, C5, C6, and C7 contained V-CV derivatives that contributed the most to the V-CV pool of seeds. C2 and C3 contained derivatives that contributed the most to the V-CV pool of the roots and shoots, respectively, independent of the variety. Finally, the derivatives in C4 contributed specifically to the V-CV pool of both vegetative organs of the variety Trumpet. These organ-associated differences were the dominant patterns observed in this dataset.

**Figure 3:**
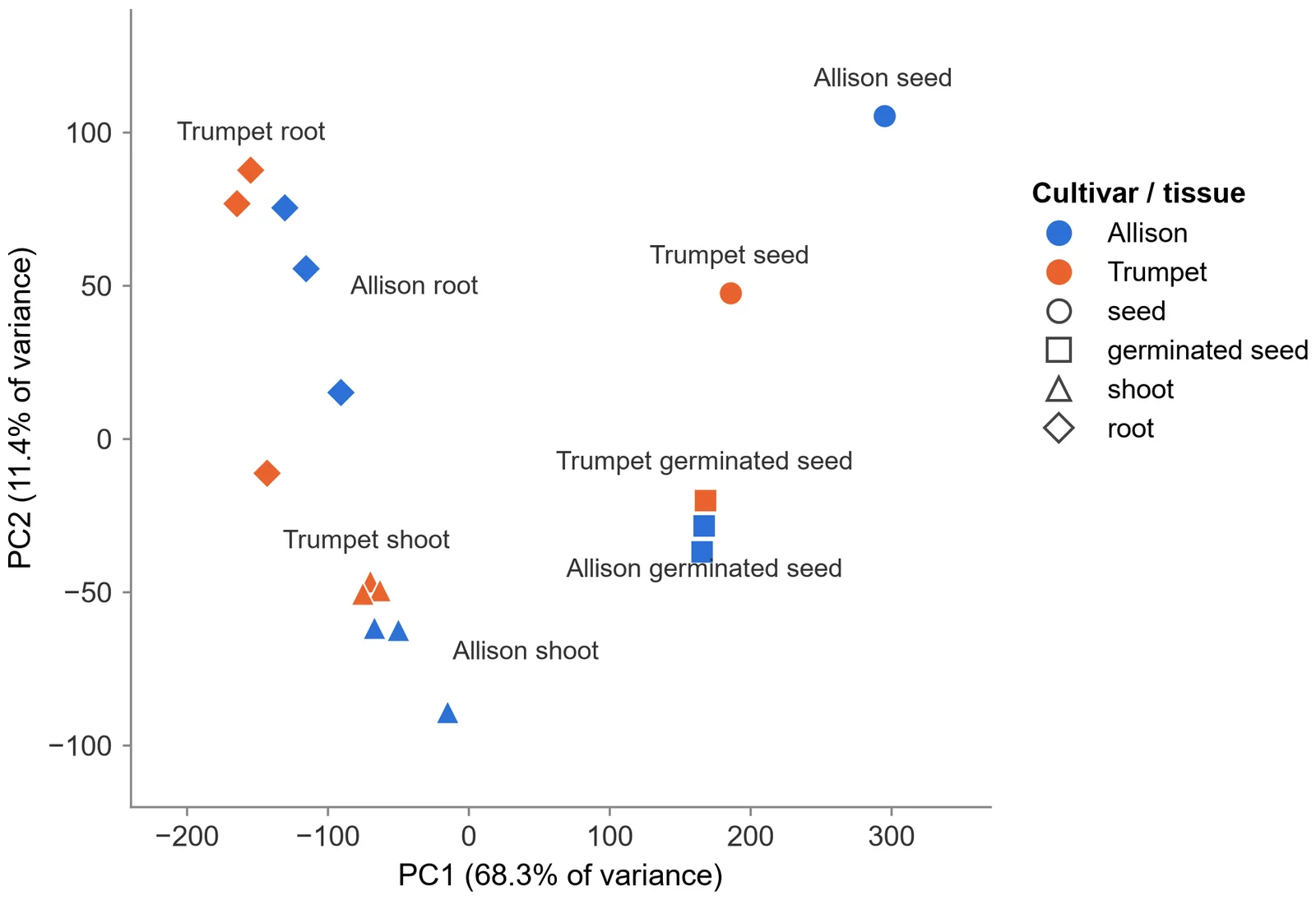
Principal component analysis (PCA) of the 160 vicine and convicine (V-CV) derivatives tentatively identified in the four organs (seeds, germinated seeds, shoot, and root) of the low-VC (LVC) variety Allison and the high-VC (HVC) variety Trumpet based on relative contribution to the V-CV pool. Samples are separated based on the organ from which the extracts are made, with PC1 (representing 68% of the variation) separating seeds (germinated or not) from vegetative organs. PC2 separates seeds (*upper left corner*) from germinated seeds and roots from shoots. Within each of the groups formed by samples from the different organs, the varietal determinant is visualized.

The observation that compounds with the same derivatizing group cluster together remains valid. Two of the seven derivatives in C6 (with the highest contribution in the seeds and germinated seeds of Allison) are (iso)valeryl V-CV, as are two isoforms of VC coumaroyl hexoside and VC caffeoyl hexoside (compounds 42, 49, 28, 31, and 30). In contrast, the hexosides of (iso)valeryl V-CV were found in C5 (compounds 48, 51, 69, and 79). The same can be observed for more complex derivatives. There were 15 compounds annotated as containing a C_10_H_14_O_3_ group. Following the annotation used in our previous study, the derivatizing group was found to be C_10_H_16_O_4_, which, based on the MS/MS spectrum, contains two carboxylic functions and is tentatively annotated as decenedioic acid. The 2-unsaturated form of which was identified in vetch (*V. sativa*) and vetch honey (Zhang et al., 2025). Compounds 141 and 148, respectively VC and CV linked to C_10_H_14_O_3_ + 432.12 amu, were found in C2. The compounds V-CV C_10_H_14_O_3_ + feruloyl(di)hexoside (compounds 142, 147, 153, and 155) were in C3, as were compounds wherein the C_10_H_14_O_3_ group links one V-CV to another (compounds 98, 99, 107, and 111). Similarly, 14 compounds were identified with a C_12_H_16_O_4_ linked to a VC and/or CV; of these, 12 were found in C5 or C7 containing compounds that were highest in Trumpet seeds, germinated or not. One of these, VC+C_12_H_16_O_4_ compound **108**, was previously recognized as a VC derivative (Valente et al., 2019). The formula for the derivatizing groups is C_12_H_18_O_5_, the only known compound with this formula in Fabaceae being a C12 acid identified in the seeds of *Phaseolus multiflorus* (Macmillan and Suter, 1967). The precursor at *m*/*z* 241.1072, with retention time of 19.23, showed a double loss of CO_2_. Based on simulated fragmentation, this compound could correspond to the C12 acid found in *P. multiflorus*.

Of the 34 compounds tentatively identified as conjugates with HCAs, 20 are conjugates with ferulic acid that are predominantly found in C2 and C3, with the highest contribution to the V-CV pool in the roots and shoots. Conjugates with the other HCAs are relatively simple, VC or CV derivatized with HCA and their hexosides, and found in clusters linked with a higher contribution in the seeds. This was not the case for the ferulic acid derivatives. V-CV feruloyl hexosides (compounds 73 and 83) were found in cluster 4, specifically contributing the most to the V-CV-pool in the shoots and roots of Trumpet. VC and CV derivatives with up to three ferulic acid groups and hexoses were identified (compounds 124 and 126 diferuloyl dihexoside derivatives, compounds 122 and 125 diferuloyl trihexoside, and compounds 151 and 154 triferuloyl dihexoside derivatives) and were found in C3. Similar compounds with the other HCAs or combinations of different HCAs were actively searched for, but not found. This dominance of ferulic acid over the other HCAs can have multiple explanations. Either the conjugating enzyme is specific for ferulic acid or, similar to what was noted above for the abundance of HCA derivatives in the seeds of the variety Sunrise, there is a higher availability of ferulic acid compared with the other HCAs. The first explanation cannot be denied nor confirmed since the conjugating enzyme is unknown. However, activated ferulic acid is abundant in the roots and shoots because of its involvement in lignification and cell wall esterification (Dos Santos et al., 2008; Buanafina and Morris, 2022), whereas activated forms of the other HCAs are less abundant. A non-selective enzyme would therefore yield predominantly feruloylated derivatives as a direct consequence of substrate availability. This generates a testable hypothesis: the composition of the V-CV derivative pool within each organ should track that of free HCAs, a test that, however, requires the absolute quantification of the derivatives.

The data presented here are descriptive, and limitations in the interpretation must be considered. First, a higher MS signal intensity for some of the derivatives than for V-CV does not mean that the derivative is more abundant than the mother compounds (Chaleckis et al., 2019). However, under certain conditions, met in the comparison of the seeds, the relative abundance of a compound in different samples can be assessed in a semi-quantitative manner (Zhou et al., 2012). The data on the seeds were acquired in a single batch from ground seeds extracted in a single experiment and were analyzed in a single Progenesis experiment. For the second part of this study, no such rigor was possible. The plant matrices were too different, which may have impacted the extraction efficiency. While this is the primary reason for selecting a comparison of the relative contribution of each derivative to the V-CV pool of the different organs and not a semi-quantitative analysis, issues related to the acquisition and treatment of the LC-MS data also need to be considered. The data were acquired on different dates, and alignment of the LC runs with Progenesis resulted in too many misalignments and errors in peak picking. When comparing the extracts from samples as diverse as in the second part of this study, the co-elution, matrix effects, or ion suppression in one of the sets of data, identified as critical issues in biomarker discovery (Chaleckis et al., 2019), limit the possibility for semi-quantitative analyses. For these reasons, the contribution of each derivative to the total pool of identified V-CV derivatives in the different organs was compared. The impact of the issues mentioned above was minimized when extracting data from individual, organ-specific Progenesis experiments as each individual Progenesis experiment was performed under conditions that allow semi-quantitative analysis. Because further identification in each of these experiments was performed using the same database, a derivative present in an extract with a high enough signal intensity will be identified. Secondly, apart from the derivatives for which the structure was determined in another study (Irmer et al., 2026), all identifications must be considered tentative based on specific mass shifts observed in the MS/MS spectra, the fragment ions for the derivatizing groups, and the presence of the fragment ions corresponding to the mother compounds at *m*/*z* 141.018 for CV and at *m*/*z* 141.042 for VC. Finally, the data presented are snapshots of the metabolome of the different organs, and factors such as active degradation and selective stability of derivatives in the different organs were not considered.

In the first part of this study, data were shown confirming the general observations previously made after profiling of the V-CV pool of the shoots and roots of faba bean plantlets (Sergeant et al., 2024). As for these organs, each variety showed a unique seed V-CV pool composition. For the seed extracts, the relative abundance of the non-V-CV compounds are in line with current knowledge on, for instance, the impact of seed germination on selected sugar oligomers and flavonoids (Nagasawa et al., 1961; Goyoaga et al., 2008; Wei et al., 2022). A recent study showed the same uniqueness in seed composition based on the protein and metabolite patterns, concluding that this is the result of the stability of cultivars obtained through breeding (Brandt et al., 2026). Clustering of the varieties based on their V-CV derivatives confirmed the LVC–HVC classification of the varieties used: in general, the MS signal intensities of the derivatives are lower in LVC than in HVC varieties. Only the variety Daisy, classified as HVC, clustered with the three LVC varieties due to the presence of three close to unique derivatives with a low MS signal intensity for the majority of the other derivatives (**Figure 2**). The difference between LVC and HVC not being limited to the presence and the abundance of VC, CV, or their derivatives is shown in **Supplementary Figure S1**. This illustrates the narrow genetic base of the LVC trait resulting in farther-ranging effects on the phytochemical profile of faba beans.

The observation of the uniqueness of the V-CV profile is expanded in the second part of this study. Organ-associated differences were the dominant pattern in the composition of the V-CV pool of the seeds, germinated seeds, shoots, and roots of a LVC and a HVC variety, with variety being a second-level discriminant (**Supplementary Figure S2**). V-CV derivatives with the same derivatizing group tended to be found in the same cluster, as was exemplified by the clustering of compounds 42 and 49, (iso)valeroyl CV and VC derivatives with a high MS signal intensity identified in different studies (Valente et al., 2019; Kowalczyk et al., 2021; Karolkowski et al., 2023; Irmer et al., 2026), with two out of seven derivatives in cluster 6. This indicates that the synthesis and/or accumulation of these derivatives is tightly controlled either by the presence of specific enzymatic activities or by the availability of the derivatizing groups, and thus genetic control. The latter appears to be the case in the seeds of the variety Sunrise, the only tannin-free, white-flowered faba bean variety in the test set. In the first cluster of **Figure 2**, which contains derivatives that are high in this variety, 20 of the 28 derivatives with HCA and VC vanillic acid were found, coinciding with the high abundance of HCAs and vanillic acid hexoside in the total metabolite profile (**Supplementary Table S3**).

All this generates a picture of a seed V-CV pool dominated by fewer derivatives toward chemically complex derivatives in the roots and shoots, with little compositional overlap between organs. This suggests that an organ-specific derivatization chemistry acting on a locally available V-CV pool and using co-localized compounds is in place, although derivatization prior to or during transport cannot be excluded.

While the data showed that each organ and each variety possess a specific V-CV profile, the implications of this specificity, physiologically or for breeding, are unclear. A recent study showed that some of the derivatives reported here contribute to the off-taste of faba bean protein isolates and thus have a deterrent activity. This study concludes that breeding of varieties that contain fewer V-CV derivatives may make faba bean protein isolates more attractive for human consumption (Irmer et al., 2026). For the mother compounds, it is known that hydrolysis of the β-glucosidic bond, which releases divicine and isouramil, initiates the redox activities of the aglycones, resulting in the depletion of glutathione (Chevion et al., 1982) and the generation of reactive oxygen species (Musci et al., 1987). As was confirmed by NMR for some derivatives (Irmer et al., 2026), and in our data confirmed when possible, all derivatizing groups are attached to the V-CV glucose, leaving the B-glucoside bond potentially accessible for hydrolysis, as in VC and CV. If this bond can indeed be hydrolyzed, the derivatives would contribute to the total amount of divicine and isouramil and, thus, to their physiological effects (Hegazy and Marquardt, 1984; Desroches et al., 1995). Whether these derivatives are hydrolyzed under the same conditions as VC and CV is not known, nor was this assessed in the current study. Studies to test this hypothesis would consist of repetitions of experiments done with the mother compounds using either the complete V-CV pool found in extracts or the synthesized V-CV derivatives. These questions can be most directly addressed by repeating the experiments described in those papers that established the link between faba bean, V-CV, and favism, for instance the work by Mager et al. published in 1965 (Mager et al., 1965). The toxicity of the V-CV derivatives can be assessed by comparing the effects of the incubation of red blood cells with a faba bean extract with the effects of a sample composed of VC and CV in the exact same quantities as present in the extract. When combined with the relative quantification of the remaining VC, CV, and the derivatives thereof, the hydrolysis of the derivatives, and thus a causal effect, can also be established. Similarly, studies such as that described by Rizzello et al. (2016) could be adapted to include V-CV derivatives. In this, the faba bean flour can be replaced by flour free of VC and CV and the effects of this flour spiked with a faba bean extract compared with that of the flour spiked with CV and VC in the quantities present in the extract.

However, the most intriguing question is why faba beans synthesize this diverse and complex arsenal of V-CV derivatives. Does the chemical diversity of derivatives provide broader protection against pests compared with what could be provided by single molecules? Furthermore, derivatization of secondary plant metabolites is known to have multiple, often unrelated roles such as transport, storage, or stabilization to avoid *in planta* toxic activities, which may also be the case for VC and CV.

## 4 Conclusion

Based on LC-MS/MS profiling, this study showed that the seeds of the tested varieties of faba bean each contain a unique set of V-CV derivatives. Furthermore, clustering of the varieties based on the MS signal intensities of the derivatives reflects the classification of the varieties into LVC or HVC, with the variety Daisy as an exception. In the current study, this HVC clusters with the LVC varieties and is characterized by three derivatives not found in the other varieties. In the second part of this study, it was shown that the composition of the V-CV pool is mainly organ-specific, while the variety of faba bean studied is a secondary factor. Together, this indicates that the biosynthesis and the accumulation of V-CV derivatives are determined by genetic regulation and that the availability of derivatizing groups may be a factor to consider. The description of these derivatives may be important to consider in future studies on the biosynthesis and biological activities of VC and CV. Seeds are currently confirmed as the main structures in which VC and CV biosynthesis is taking place. However, the studies cited above found that the amounts of VC and CV in seeds do not account for the total quantities of these compounds in other organs. The inclusion of V-CV derivatives in the mass balance between different organs may contribute to the discovery of the biosynthetic pathways and the organs in which these compounds are synthesized. Furthermore, current knowledge relates the biological activities of V-CV to the hydrolysis of the glucosidic bond, liberating divicine and isouramil. While unknown, and not addressed in this study, the hydrolysis of this bond in the derivatives may contribute to the amount of divicine and isouramil that is released. For the moment, these questions remain unanswered. However, considering that not all divicine/isouramil is present as VC or CV may result in new knowledge on these compounds and lead to new breeding targets or influence current breeding efforts.

## Data Availability

The original contributions presented in the study are included in the article/**Supplementary Material**. Further inquiries can be directed to the corresponding author.

## Supplementary material

Supplementary Figure 1 PCA based on the total seed LC-MS/MS profile of ten varieties of faba bean. LC-MS/MS runs of extracts from ten varieties of faba bean were uploaded in Progenesis, known contaminants and compounds without MS/MS spectrum excluded, resulting in 2824 features used for PCA. The LVC varieties Futura, Allison and Callas cluster together while the variability among the HVC varieties is larger.

Supplementary Figure 2 Heatmap analysis of the 160 tentatively identified V-CV derivatives based on the contribution of each derivative to the total V-CV MS-signal intensity in each extract. Seed samples, in columns, are separated from the other samples, and germinated seeds from the vegetative organs. Within each group, representing an organ, samples are further clustered based on the variety.

Supplementary Table 1 Agronomic and yield data for the ten varieties of faba bean used in this study. Field performance and disease susceptibility data are collected following faba beans variety trails in organic farming for Luxembourg by the Institute for Organic Agriculture and Agroecology Luxembourg a.s.b.l. ((IBLA). The faba beans were sown in March 2023 and harvested in July 2023 in Folkendange canton Diekirch (nearest weather station Ettelbruck) (https://ibla.lu/_res/uploads/2024/01/2024_01_10_Methodenhandbuch_final.pdf, https://ibla.lu/_res/uploads/2024/01/2024_01_10_Endbericht_Sortenpruefung-Koernerleguminosen_2023_final.pdf).

Supplementary Table 2 LC-MS data for the 104 derivatives considered for the analysis of the V-CV diversity in ten varieties of faba bean. Reference; the number of the compounds in **Figure 2**; Raw abundance; the raw MS-signal intensities as extracted from Progenesis.

Supplementary Table 3 LC-MS data for some selected phenolic compounds in the extracts from seeds from ten varieties of faba bean.

Supplementary Table 4 LC-MS data for the 160 derivatives considered for the analysis of the V-CV diversity in seed, germinated seeds, shoots and roots of plantlets from the LVC variety Allison and the HVC variety Trumpet. Reference and Cluster; refer respectively to the identifiers found on the right of the heatmap in **Supplementary Figure S2** and the clustering obtained, Raw abundance; the raw MS-signal intensity as extracted from Progenesis, the *per mille* contribution of the derivatives to the V-CV pool of each sample was used for heatmap analysis as the result of which is represented in **Supplementary Figure S2**.
